# A novel technique based on *in vitro* oocyte injection to improve CRISPR/Cas9 gene editing in zebrafish

**DOI:** 10.1038/srep34555

**Published:** 2016-09-29

**Authors:** Shao-Lin Xie, Wan-Ping Bian, Chao Wang, Muhammad Junaid, Ji-Xing Zou, De-Sheng Pei

**Affiliations:** 1 College of Marine Sciences, South China Agricultural University, Guangzhou, 510642, China; 2 Chongqing Institute of Green and Intelligent Technology, Chinese Academy of Sciences, Chongqing, 400714, China; 3 University of Chinese Academy of Sciences, Beijing 100049, China

## Abstract

Contemporary improvements in the type II clustered regularly interspaced short palindromic repeats/CRISPR-associated protein 9 (CRISPR/Cas9) system offer a convenient way for genome editing in zebrafish. However, the low efficiencies of genome editing and germline transmission require a time-intensive and laborious screening work. Here, we reported a method based on *in vitro* oocyte storage by injecting oocytes in advance and incubating them in oocyte storage medium to significantly improve the efficiencies of genome editing and germline transmission by *in vitro* fertilization (IVF) in zebrafish. Compared to conventional methods, the prior micro-injection of zebrafish oocytes improved the efficiency of genome editing, especially for the sgRNAs with low targeting efficiency. Due to high throughputs, simplicity and flexible design, this novel strategy will provide an efficient alternative to increase the speed of generating heritable mutants in zebrafish by using CRISPR/Cas9 system.

Loss-of-function is an important approach for *in vivo* functional study of a gene of interest in transgenic animals. The RNA-guided clustered regularly interspaced short palindromic repeats (CRISPR)/Cas9 nuclease system has been proved to be another precise and efficient genome editing technique after zinc-finger nucleases (ZFNs) and transcription activator like effector nucleases (TALENs) in recent years^1^,^2^,^3^,^4^,^5^. This promising tool has been successfully applied for genome editing in many model organisms, including zebrafish, mice, dog, pig, *Drosophila, C. elegans*, and primates, *etc*^6^,^7^,^8^,^9^,^10^,^11^,^12^. No matter which gene editing tool is used, low efficiencies of genome editing and germline mutant transmissions usually lead to labor intensive and time consuming screening work to acquire high-throughputs. For instance, zebrafish founders carried 64–81% *ntl* mutations by ZFNs technique, but among them only 5–32% *ntl* founders were resulted in heritable germline mutations^13^. Although TALENs have been proved to be a powerful technique for genome editing, the mutation frequency of TALENs still has room to improve^14^. Recently, CRISPR/Cas9 system has emerged as an efficient alternative for genome editing due to high-throughputs and broad spectrum of applications. However, the efficiency of germline transmission by CRISPR/Cas9 needs further improvements. For example, Hruscha *et al*. reported 50.5% mutation frequencies in zebrafish founder embryos, but only about 11% of mutations inherited to their progeny^15^. Varshney *et al*. obtained an average germline transmission rate of 28% from 162 target sites in zebrafish genome^16^.

To improve the efficiencies of genome editing and germline transmission, researchers explored many different approaches. Maruyama *et al*. promoted the homology-directed repair (HDR) and increased the genome editing efficiency of CRISPR/Cas9 system by inhibiting non-homologous end joining(NHEJ)^17^. Zhang *et al*. established a highly efficient CRISPR mutagenesis system by introducing microhomology-mediated end joining (MMEJ) with very short (approximately 35 bp) homology arms^18^. In addition, Yan and Schwartz used optimal promoters to drive the expression of Cas9 and sgRNAs to obtain highly efficient genome modifications^19^,^20^. In addition, Carrington *et al*. reported an easy fluorescent PCR-based method to pre-screen sgRNAs for target-specific activity^21^. Moreover, Renaud *et al*. also improved genome editing efficiency by using phosphorothioate-modified oligonucleotides^22^. Fujii *et al*. and Sung *et al*. found that Cas9 protein induced genome modifications more rapidly than Cas9 capped RNA^23^,^24^ and codon-optimized Cas9 protein improved higher knockout efficiency than original one^25^,^26^. Nevertheless, the subsequent screening for germline transmission of induced mutations could be time-consuming and labor intensive for the sgRNAs with low targeting efficiencies^15^,^27^. In 2014, Dong *et al*. reduced the screening time for mutants by labelling the zebrafish primordial germ cells^28^. However, the improvement for the specific targeting efficiency remained below expectations.

A valuable technique of ovum preservation has been successfully applied in mammals^29^,^30^,^31^, including humans^32^,^33^,^34^. Recently, the preservation of fish oocytes *in vitro* was also reported^35^,^36^. Goetz and his colleagues used modified cortland medium to store trout eggs for at least 2 days^37^. Ubilla *et al*. reported that *in vitro* storage of rainbow trout eggs in their coelomic fluid for 96 h caused a decline in the quality of eggs and reduced their capacity to develop into larvae^38^. The preferable preservation technology of zebrafish oocytes was established in 2008 by Seki *et al*., which contained 90% Leibovitz L-15 medium at pH 9.0, containing 0.5 mg mL^−1^ BSA and 1 ug mL^−1^ 17α-20β-Dihydroxy-4 Pregnen-3-one (DHP). In their findings, they kept the zebrafish oocytes alive from stage III to mature *in vitro*, nevertheless the hatching rate remained low^39^,^40^.

Here, we reported a modified *in vitro* oocyte storage strategy to increase the efficiency of genome editing by CRISPR/Cas9 system in zebrafish. This novel technique with *in vitro* oocyte injection prior to their storage improved the efficiencies of gene targeting and germline transmission. Contrary to the conventional genome editing approaches, this method utilized the inherent translation system to impel the oocytes to produce Cas9 proteins in advance, which greatly increased the efficiency of genome editing. Undoubtedly, it also improved the efficiency of germline transmission. Therefore, our novel technique will increase the speed of generating heritable mutants of zebrafish using CRISPR/Cas9 system.

## Materials and Methods

### Ethics Statement

All experiments in this study were in accordance with the “Guide for the Care and Use of Laboratory Animals” (Eighth Edition, 2011. ILARCLS, National Research Council, Washington, D.C.) and were approved by the Animal Care and Use Committee of Chongqing in China and by the Institutional Animal Care and Use Committee of Chongqing Institute of Green and Intelligent Technology, Chinese Academy of Sciences (Approval ID: ZKCQY0068).

### Zebrafish husbandry

Adult fish and embryos of AB strains were raised and maintained as described by Westerfield *et al*.^41^,^42^. Fish were kept in the automatic water cycle system on a 14 h light: 10 h dark period. The adult female and male zebrafish were maintained at 28 °C in separate tanks and mated once a week.

### CRISPR guide RNAs design and Cas9 capped RNA synthesis

Cas9 target sites were selected in exons across the genome and designed with an online tool, ZIFIT Targeter (http://zifit.partners.org/ZiFiT/ChoiceMenu.aspx). The plasmid pXT7-Cas9 was linearized by *Xba*I^6^. Cas9 capped RNA was synthesized using Ambion mMESSAGE mMACHINE T7 Transcription Kit (Ambion, USA). The sgRNAs transcription templates were prepared by PCR with T7-targetsite-F primers and a universal reverse primer gRNA-R (Table 1)^43^. The sgRNAs PCR products were purified by TIANquick Midi Purification Kit (TIANGEN, China) and then transcribed with MAXIscript T7 Kit (Ambion, USA). RNAs were purified by MicroElute RNA Clean-Up Kit (OMEGA, USA). The RNA quality and concentration were analyzed by electrophoresis and the NanoDrop2000 spectrophotometer (Thermo Fisher Scientific, France). The efficiencies of sgRNAs were examined by the sgRNAs activity detection kit *in vitro* (Viewsolid Biotech, China).

### *In vitro* oocyte storage

The general method of *in vitro* oocyte storage is adapted from Seki *et al*.^39^ and Nair *et al*.^40^. The oocyte storage medium was 90% Leibovitz’s L-15 medium with L-glutamine (Gibco) and 0.5 mg mL^−1^ bovine serum albumin (BSA, Sigma-Aldrich), and then pH was adjusted to 9.0 with 10N NaOH. Before oocyte collection, adult zebrafish females and males were maintained in separate spawn tanks overnight. Adult zebrafish females with swollen and elastic abdomen were chosen. Before squeezing the oocytes, zebrafish females were treated with 0.2 mg mL^−1^ ethyl 3-aminobenzoate methanesulfonate salt, then gently pressed the abdomen under the microscope. Oocytes were harvested from 1–2 females and scattered in the oocyte storage medium.

### Oocyte micro-injection

**Figure 1:**
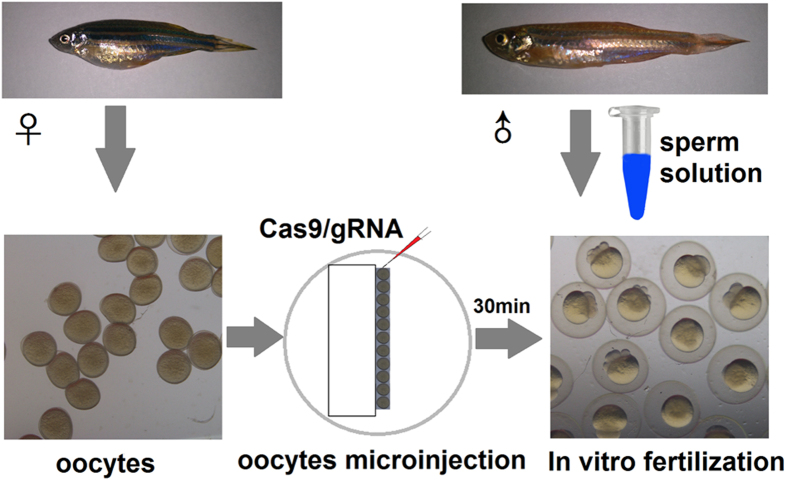
The schematic diagram of microinjection and oocyte storage.

The mature oocytes were collected for micro-injection by using a micro-injector (Eppendorf, Germany). The injected reagents were diluted in 0.2 M KCl. The final concentrations of Cas9 RNAs and sgRNAs were 300 ng μL^−1^ and 30~50 ng μL^−1^, respectively. One nL solution containing 300 pg Cas9 capped RNA and 30~50 pg sgRNAs was micro-injected into 1-cell embryo. The oocytes were arranged along the edge of a glass slide in oocyte storage medium before micro-injection (Fig. 1). For gene knock-in experiment, Cas9 capped RNAs, *mc4r* sgRNAs and the single-stranded *mloxP* donor DNAs (Table 1) were micro-injected into zebrafish oocytes. After completion of micro-injection, the oocytes were cultured in the storage medium to avoid light for 30 min.

### *In vitro* fertilization

Injected oocytes were fertilized after 30 min. Sperm solution was produced by shearing the testes from two AB male fish and grinding in 200~300 μL cortland solution (NaCl 7.25 g L^−1^; KCl 0.38 g L^−1^; CaCl_2_ 0.162 g L^−1^; NaHCO_3_ 1.0 g L^−1^; NaH_2_PO_4_ 0.41 g L^−1^; MgSO_4_ 0.23 g L^−1^; glucose 1.0 g L^−1^). The quality of sperm was judged by the color of the sperm solution. The color of mature sperm was milky white. The cortland solution was kept on ice for at least 1 h before use. During this experiment, the sperm solution was kept on ice all the time. The storage medium was removed from the culture dish, and 20~30 μL sperm solution was added directly on the top of the oocytes, followed by a few drops of fresh water with a plastic dropper. After 1~2 min, the culture dish was washed gently with fresh water.

### Genomic DNAs isolation

Genomic DNAs were extracted by using the CTAB extraction method^44^. For the DNAs extraction of embryos and oocytes, one embryo and five oocytes were incubated in 40 μL and 60 μL lysis solution (100 mM Tris-Hcl pH 8.5, 0.5 M EDTA, 10% SDS, 5 M NaCl, 20 mg mL^−1^ Proteinase K) at 55 °C for 2 h. For adult zebrafish DNAs extraction, partial tails were lysed in 100 μL lysis solution, and incubated at 55 °C for 3 h. Then, DNAs were purified by chloroform, isopropanol and 75% ethanol. Finally, 20~30 μL double distilled water was added into each sample.

### Gene editing assay and sequencing analysis

The sgRNAs binding site and the primers used for T7E1 assay are shown in Table 1. PCR mixture was prepared according to the manual of TransFast Taq DNA Polymerase (Transgen Biotech, China). The PCR program was set as: 95 °C 2 min, 35 cycles of (95 °C 5 s, *mc4r* 52 °C, *mpv17* 55 °C, *mstna* 55 °C, *mc3r* 55 °C, *mrap2b* 60 °C, 15 s, and 72 °C 10 s), and a final extension at 72 °C for 5 min (BIO-RAD, Singapore). 8.5 μL PCR products were added into 1 μL T7 endonuclease I buffer (Viewsolid Biotech, China) to perform an annealing process (95 °C for 5 min, 95 °C to 75 °C ramping at 0.1 °C s^−1^, 75 °C to 16 °C ramping at max rate and holding at 16 °C for 2 min) for heteroduplex formation. After annealing, products were treated with 0.5 μL T7 endonuclease I for 15 min at 37 °C, and analyzed on 1.5% agarose gels. Gels were observed and analyzed by using a gel-imaging system (JUNYI, Beijing, China). To detect *mloxP* knock-in, larvae DNAs at 72 hpf were isolated and PCR was performed with *mc4r* T7E R matching the *mc4r* sequence and *mloxP R* matching the sequence of the *mloxP* donor sequence (Table 1). To further confirm the success of gene editing, about 2 μL above all PCR products were cloned into the pMD-19T vector (Takara, Japan) for sequencing analysis.

### Statistical analysis

For statistical analysis, two-tailed unpaired Student’s t-test was performed by using SPSS software^45^. P values < 0.05 were considered statistically significant. Each experiment was performed independently for three times.

## Results

### An effective storage medium for oocyte preservation

**Figure 2:**
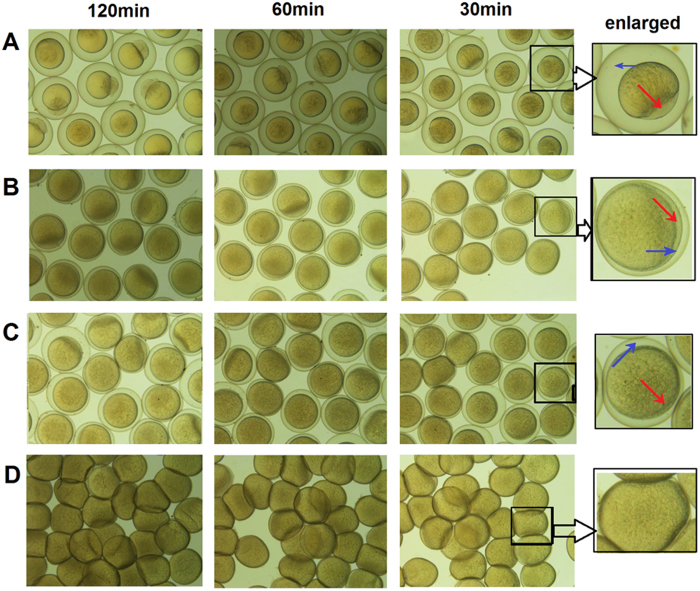
The preservation effects of three different oocyte storage media. (**A**) Fresh water, (**B**) Hepes-cortland medium, (**C**) 90% L-15 medium without BSA at pH 9.0, (**D**) 90% L-15 medium with BSA at pH 9.0. The storage time in three oocyte storage media was 30 min, 60 min and 120 min, respectively. The cavities between the shell and vitelline membrane and the animal pole were indicated with red and blue arrows.

The oocyte preservation and *in vitro* maturation have been investigated for many years. On the basis of previous findings, we compared the effects of three oocytes storage media: hepes-cortland medium, 90% L-15 medium at pH 9.0 with BSA, and without BSA. Due to the difficulty in obtaining the coelomic fluid of zebrafish, oocyte preservation in coelomic fluid was not suitable here. Thus, we collected oocytes and stored them in three different preservation media that are mentioned above. The results showed that the mature oocytes stored in 90% L-15 medium with BSA at pH 9.0 remained stable after 2 h. The hepes-cortland medium showed almost the same effect as the 90% L-15 medium without BSA at pH 9.0 and induced the formation of a cavity between the oocyte shell and vitelline membrane (Fig. 2).

### The hatching rates *in vitro*

**Figure 3:**
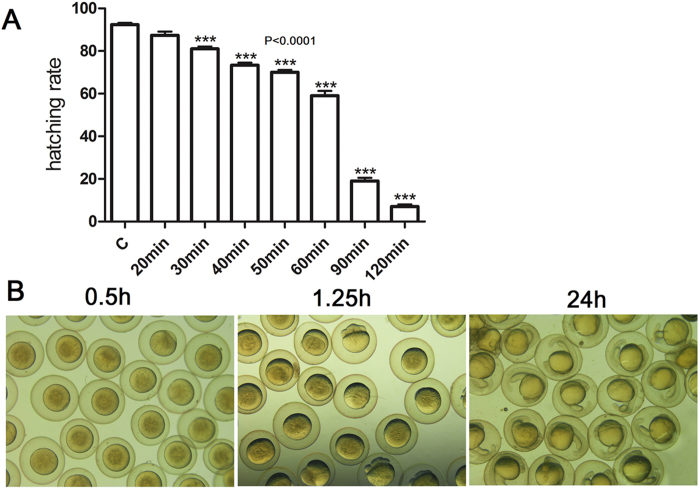
Effects of storage time on hatching rates and embryonic development in 90% L-15 medium with BSA at pH 9.0. (**A**)The relationship between the hatching rate and the storage time *in vitro*. The oocytes were stored in oocyte storage medium for different time and then fertilized *in vitro*. The hatching rates were recorded after 72 h. (**B**) The embryos developed normally when oocytes were stored for 30 min *in vitro*. The oocytes were stored in oocyte storage medium for 30 min and then fertilized *in vitro*. The embryonic development were observed from 0.5 h to 24 h.

To find a suitable storage time, the effects of storage time on hatching rates were investigated. The results showed that there was a significantly decreasing trend for oocyte hatching rates with increase in oocytes storage time *in vitro* (Fig. 3). The hatching rates dramatically declined, when the storage time was longer than 60 min (Fig. 3A). However, the egg hatching rates were more than 80%, if the storage time was less than 30 min (Fig. 3B). As expected, the oocytes stored in hepes-cortland medium and 90% L-15 medium without BSA failed to fertilize *in vitro*. In addition, a longer storage time resulted in a higher deformity rate in the 90% L-15 medium with BSA at pH 9.0 (data not shown). Our results indicated that Seki’s medium was conducive to oocytes storage, but DHP was unnecessary. However, BSA was proved as a critical component for the oocytes storage medium.

### Translational capacity of mature oocytes

**Figure 4:**
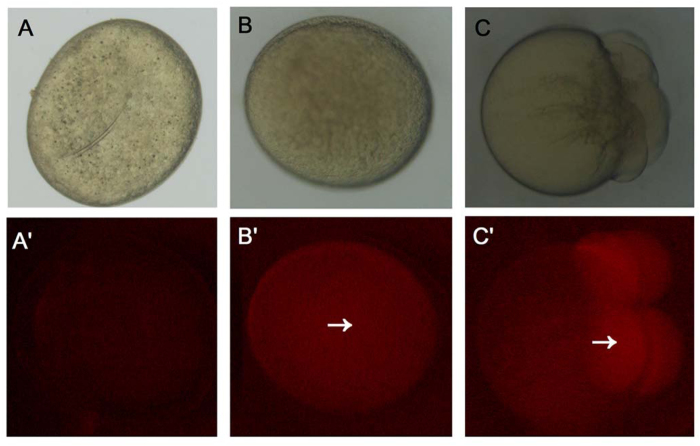
The translational capacity of mature oocytes. (**A**) Oocytes were stored for 2 h *in vitro* without injecting mCherry capped RNAs. (**B**) Oocytes were stored for 2 h *in vitro* and micro-injected with mCherry capped RNAs. (**C**) Fertilized eggs at 1 cell stage were micro-injected with mCherry capped RNAs after 2 h. The red fluorescence was indicated with white arrows.

To verify the translational capacity of mature oocytes, mCherry capped RNAs were micro-injected into mature oocytes and the oocytes were cultured in the storage medium. The red fluorescence was observed after 2 h incubation (Fig. 4). To elucidate whether the Cas9-sgRNAs complexes efficiently worked in zebrafish oocytes or not, the Cas9 capped RNAs and *mc4r* or *mpv17* sgRNAs were micro-injected into mature oocytes and incubated in the storage medium for 30 min or more, then the PCR-based T7E1 assay was performed. However, there was no mutation detected in the oocytes genome (data not shown). Thus, we speculated that the genome DNAs of zebrafish oocytes had a high affinity between histones and DNA backbone, which prevented Cas9 protein and target sgRNAs from binding with genomic DNAs to initiate the cleavage activity of CRISPR-Cas9 complex.

### Toxicity assay of Cas9 capped RNAs and sgRNAs

**Figure 5:**
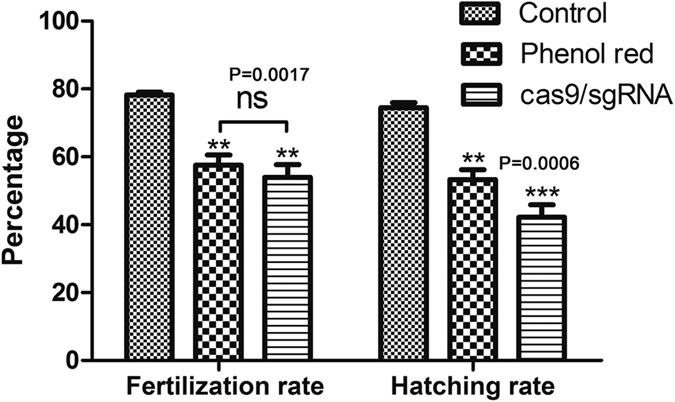
Toxicity assay of oocytes injected with Cas9 capped RNAs and sgRNAs. The control groups were oocytes stored *in vitro* for 30 min without injection. The groups of phenol red and Cas9/sgRNAs were injected with phenol red and Cas9/sgRNAs, respectively.

To test the toxicity of Cas9 capped RNAs and sgRNAs in the oocytes, the fertilization rate and hatching rate were statistically analyzed after injecting Cas9 capped RNAs/sgRNAs or phenol red. The results revealed that the toxicity of Cas9 capped RNAs/sgRNAs was very low, compared to phenol red injected groups. However, the fertilization and hatching rates were significantly lower than the control group (Fig. 5 and Table S1), indicating that the injection caused mechanical damage to the oocytes. In addition, there was no significant difference observed in fertilization rates between Cas9 capped RNAs/sgRNAs groups and phenol red groups, albeit their hatching rates were significantly different (Fig. 5 and Table S1). About eight percent of embryos were observed with developmental deformity and were unable to hatch properly after injecting Cas9 capped RNAs/sgRNAs.

### Improving the efficiency of gene knock-in in zebrafish founder

**Figure 6:**
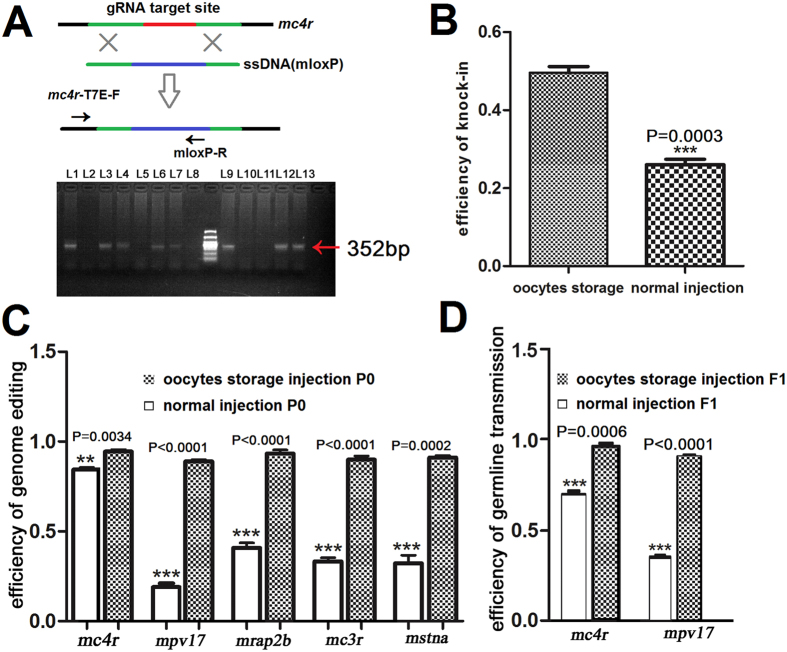
The efficiencies of gene knock-in and knock-out. (**A**) The schematic diagram of knocking *mloxP* into *mc4r* locus. A single-stranded oligo contained a *mloxP* site (blue) and 27 nt left and right homology arms (green). The primers for PCR detection were marked as black arrows. The positive fragments with *mloxP* were marked with red arrows. (**B**) The knock-in efficiencies of *mloxP* both in the oocytes storage injection groups and normal injection groups. The numbers of embryos for detection and knock-in in each experiment were shown in Table S4. (**C**) The efficiencies of genome editing of *mc4r, mpv17, mstna, mc3r* and *mrap2b* were measured by T7E1 assay and sequencing analysis. (**D**) The efficiencies of germline transmission of *mc4r* and *mpv17* were also measured by T7E1 assay and sequencing analysis in zebrafish F_1_ generation. Three batches of F_1_ embryos with different numbers (17~28) were performed both in the oocytes storage injection groups and in the normal injection groups. Each experiment was repeated for three times independently.

Although gene knock-in was successfully performed by ZFN, TALEN and Cas9 in zebrafish embryos^4^,^46^,^47^, the efficiency of gene knock-in was unsatisfactory. To test whether the current method of oocytes storage could enhance the efficiency of gene knock-in or not, a *mloxP* knock-in experiment was performed. The results were quite promising and showed that a single-stranded oligo DNA as a donor containing a *mloxP* site^6^ and 27 nt homology arms on both ends was effectively knocked into *mc4r* locus (Fig. 6A and Fig. S1B). The size of PCR products was 352 bp as expected (Fig. 6A). To further confirm the insertion of *mloxP* into *mc4r* locus, the PCR products were amplified from the tested embryo and sequenced. As shown in Fig. S1, *mloxP* were successfully knocked into *mc4r* locus both by the normal and oocytes storage injection. Moreover, the efficiency of gene knock-in was successfully improved by 49.6% in the oocytes storage injection groups, compared to 26% in the normal injection groups (Fig. 6B and Table S2).

### Enhancing the efficiency of gene knock-out in zebrafish founder

Zebrafish *mc4r, mpv17, mstna, mc3r* and *mrap2b* founders were selected to evaluate the efficiency of genome editing by using this novel technique based on *in vitro* oocyte injection and storage. Five sgRNAs were designed, which targeted *mc4r, mpv17, mstna, mc3r* and *mrap2b*, respectively. The results showed that 5 target genes were efficiently knocked out by using this technique (Figs S2 and S3). The mutation rates of those 5 genes (*mc4r, mpv17, mstna, mc3r* and *mrap2b*) were 94.4%, 88.9%, 91.1%, 90.0% and 93.3%, respectively. Compared to the conventional 1-cell micro-injection, this technique significantly improved the efficiency of genome editing in zebrafish founder. Micro-injection of sgRNAs and Cas9 capped RNAs into 1-cell embryos by using a CRISPR/Cas9 system achieved 86.7%, 18.9%, 32.2%, 33.3% and 40.7% mutation rates for *mc4r, mpv17, mstna, mc3r* and *mrap2b*, respectively (Fig. 6C and Table S3).

### Improving the efficiencies of germline transmission in the offspring

To test the efficiencies of germline transmission, *mc4r* and *mpv17* genome DNAs were extracted from F_1_ generation and T7E1 assay and sequencing analysis were performed. Results showed that the efficiencies of germline transmission for *mc4r* and *mpv17* mutations were 96.7% and 91% (Fig. 6D and Table S4), which were significantly higher than the common CRISPR/Cas9 system (70% and 35.2%).

## Discussion

Here, a novel technique based on *in vitro* oocyte injection was developed and optimized to improve the efficiencies of genome editing and germline transmission in zebrafish by using a CRISPR/Cas9 system. Previously, Nakajima and Yaoita reported that the gene editing activity of TALEN was improved by injecting TALEN capped RNAs into the oocytes of *Xenopus laevis*^48^. However, little is known about translational capacity of mature oocytes in zebrafish by using a CRISPR/Cas9 system.

Chang *et al*. reported that the CRISPR/Cas9 nuclease generated site-specific cleavage (~35%) by editing three genes in zebrafish^6^. Hwang *et al*. reported that the mutation frequencies for ten genes induced by CRISPR/Cas9 nuclease were low in zebrafish, and the highest mutation was only 59.4%^49^. The low efficiencies of genome editing and germline transmission are the common issues confronted with many researchers. To solve these problems, researchers developed and evaluated different approaches. In this study, the rates of genome editing and germline transmission for all the replicates were more than 90%, which indicated that the micro-injection of Cas9 capped RNAs into oocytes could reduce the time for Cas9 translation, and subsequently improve the probability of mutations transferred to the next generation.

Our findings are consistent with the results reported by Fujii *et al*.^23^. Gagnon *et al*. revealed that the direct injection of Cas9 protein and sgRNAs complex into zebrafish eggs strikingly increased the mutagenic activity, but the efficiency of germline transmission was not discussed^50^. Our current findings are entirely different from the results using the direct injection of Cas9 protein/sgRNAs complex. Since the commercial Cas9 protein purified from bacteria lacked post-translational modifications, Cas9 proteins antecedently expressed in oocytes exhibited higher enzyme activity than the Cas9 protein derived from bacteria. Thus, this novel technique based on *in vitro* oocyte injection can improve the efficiencies of knock-out, knock-in and germline transmission by using a CRISPR/Cas9 system. It’s worth mentioning that an increasing efficiency of knocking *mloxP* into *mc4r* locus was achieved in this study, but whether the knocking-in efficiency was correlated with that of germline transmission deserves further studies.

The choices of effective storage media and appropriate storage time were the critical factors to affect the probability of the successful oocytes storage *in vitro*. The oocytes of freshwater fish usually develop the cavity between the shell and vitelline membrane due to osmotic phenomenon. In this study, the cavity forming effects of three oocytes storage media were compared. However, 90% L-15 medium at pH 9.0 with BSA was the only medium for inhibiting the formation of cavity. The cavity between the oocyte shell and vitelline membrane can hinder the oocytes’ fertilization, which was confirmed by hepes-cortland medium and 90% L-15 medium without BSA. Generally, the hatching rates of oocytes are closely associated with the storage time *in vitro*. Seki *et al*. obtained 12% hatching rates by making the zebrafish oocytes matured *in vitro* from stage III^35^. In this study, relatively longer storage time was resulted in higher oocytes deformity rates. Therefore, 30 min storage time was appropriate to improve the hatching rate and the translation of Cas9 protein in zebrafish oocytes.

In conclusion, a novel technique based on *in vitro* oocyte injection and storage was developed and evaluated in this study. The combination of these two techniques was confirmed to improve the efficiencies of genome editing and germline transmission in zebrafish by using a CRISPR/Cas9 system. This novel strategy will reduce the unnecessary labor intensive screening and will significantly increase the speed of generating heritable mutants in zebrafish.

## Additional Information

**How to cite this article**: Xie, S.-L. *et al*. A novel technique based on *in vitro* oocyte injection to improve CRISPR/Cas9 gene editing in zebrafish. *Sci. Rep.* **6**, 34555; doi: 10.1038/srep34555 (2016).

## Supplementary Material

Supplementary Information

## Figures and Tables

**Table 1:** The sequences of primers and target spacers used in this study.

Primer Description	Sequence (5′ to 3′)
T7-targetsite-F	TAATACGACTCACTATAG-targetsite-GTTTTAGAGCTAGAAATAGC
gRNA-R	AGCACCGACTCGGTGCCAC
*mc4r* gRNA target spacer	GGGGGTGTTTGTGGTGTGCT
*mc4r*-T7E-F	GACCGCTACATCACAATCT
*mc4r*-T7E-R	TTGGCTTCTGAAGGCATAT
*mpv17* gRNA target spacer	GGGTCTTTGGAGATCTTATC
*mpv17*-T7E-F	CCGTTTGTCATAATGTGG
*mpv17*-T7E-R	CTGCTTAGGGAGGTTTCT
*mstna* gRNA target spacer	GGTTGGCTCCTCAGCTGGT
*mstna* -T7E-F	TTGCAGCTCAAGCCATCA
*mstna* -T7E-R	GGTCACAGCCAGGTCATT
*mrap2b* gRNA target spacer	GCTGGAAGTGGGCGGGTCTC
*mrap2b* -T7E-F	AATAGAGAGGGAAGAGGGCGA
*mrap2b* -T7E-R	AGAAAGTCGTCATGGCCGAG
*mc3r* gRNA target spacer	GACCGTACGCAGAGCTCTGG
*mc3r* -T7E-F	TTCTCACCCTGGGCATCG
*mc3r* -T7E-R	GAGTCGCCATAAGCACTA
T7mCherry-F	TAATACGACTCACTATAGGGCTAGCGCTACCGGTCGC
T7mCherry-R	AAAAAACCTCCCACACCTCCCCCTG
*mc4r mloxP* donor	GGGCGCCATTACCATTACTATACTACTATAAGCTTCGTATAGCATACATTATAGCAATTTATGGGCGCCCTTTTTCTTGCACCTCATCC
*mloxP F*	ATAAGCTTCGTATAGCATACATTATAGCAATTTA
*mloxP R*	ATAAATTGCTATAATGTATGCTATACGAAGCTTAT
*mloxP*- KI-JC-F	GCACCACTGTTCTCATCTGCCTCAT
*mloxP*- KI-JC-R	TGAGGATGAGGTGCAAGAAAAAGGG

## References

[b1] Ran F. A. . Genome engineering using the CRISPR-Cas9 system. Nature protocols 8, 2281–2308, doi: 10.1038/nprot.2013.143 (2013). 24157548 PMC3969860

[b2] Cong L., Ran F. A., Cox D., Lin S., Barretto R., Habib N., Hsu P. D., Wu X., Jiang W., Marraffini L. A. & Zhang F. Multiplex genome engineering using CRISPR/Cas systems. Scientific reports 339, 23, doi: 10.1126/science.1231143 (2013). PMC3795411 23287718

[b3] Remy S. . Zinc-finger nucleases: a powerful tool for genetic engineering of animals. Transgenic research 19, 363–371, doi: 10.1007/s11248-009-9323-7 (2010). 19821047

[b4] Bedell V. M. . *In vivo* genome editing using a high-efficiency TALEN system. Nature 491, 114–118, doi: 10.1038/nature11537 (2012). 23000899 PMC3491146

[b5] Feng Y., Zhang S. & Huang X. A robust TALENs system for highly efficient mammalian genome editing. Scientific reports 4, 3632, doi: 10.1038/srep03632 (2014). 24407151 PMC3887383

[b6] Chang N. . Genome editing with RNA-guided Cas9 nuclease in zebrafish embryos. Cell research 23, 465–472, doi: 10.1038/cr.2013.45 (2013). 23528705 PMC3616424

[b7] Terao M. . Utilization of the CRISPR/Cas9 system for the efficient production of mutant mice using crRNA/tracrRNA with Cas9 nickase and FokI-dCas9. Experimental animals/Japanese Association for Laboratory Animal Science, doi: 10.1538/expanim.15-0116 (2016). PMC4976241 26972821

[b8] Zou Q. . Generation of gene-target dogs using CRISPR/Cas9 system. Journal of molecular cell biology 7, 580–583, doi: 10.1093/jmcb/mjv061 (2015). 26459633

[b9] Su Y. H., Lin T. Y., Huang C. L., Tu C. F. & Chuang C. K. Construction of a CRISPR-Cas9 System for Pig Genome Targeting. Animal biotechnology 26, 279–288, doi: 10.1080/10495398.2015.1027774 (2015). 26158460

[b10] Lee J. S. . RNA-guided genome editing in Drosophila with the purified Cas9 protein. G3 4, 1291–1295, doi: 10.1534/g3.114.012179 (2014). 24875628 PMC4455777

[b11] Friedland A. E. . Heritable genome editing in C. elegans via a CRISPR-Cas9 system. Nature methods 10, 741–743, doi: 10.1038/nmeth.2532 (2013). 23817069 PMC3822328

[b12] Niu Y. . Generation of gene-modified cynomolgus monkey via Cas9/RNA-mediated gene targeting in one-cell embryos. Cell 156, 836–843, doi: 10.1016/j.cell.2014.01.027 (2014). 24486104

[b13] Doyon Y. . Heritable targeted gene disruption in zebrafish using designed zinc-finger nucleases. Nature biotechnology 26, 702–708, doi: 10.1038/nbt1409 (2008). PMC2674762 18500334

[b14] Bedell V. M. . *In vivo* genome editing using a high-efficiency TALEN system. Nature 491, 114–118, doi: 10.1038/nature11537 (2012). 23000899 PMC3491146

[b15] Hruscha A. . Efficient CRISPR/Cas9 genome editing with low off-target effects in zebrafish. Development 140, 4982–4987, doi: 10.1242/dev.099085 (2013). 24257628

[b16] Varshney G. K. . High-throughput gene targeting and phenotyping in zebrafish using CRISPR/Cas9. Genome research 25, 1030–1042, doi: 10.1101/gr.186379.114 (2015). 26048245 PMC4484386

[b17] Maruyama T. . Increasing the efficiency of precise genome editing with CRISPR-Cas9 by inhibition of nonhomologous end joining. Nature biotechnology 33, 538–542, doi: 10.1038/nbt.3190 (2015). PMC4618510 25798939

[b18] Zhang C., Meng X., Wei X. & Lu L. Highly efficient CRISPR mutagenesis by microhomology-mediated end joining in Aspergillus fumigatus. Fungal Genetics and Biology 86, 47–57, doi: 10.1016/j.fgb.2015.12.007 (2016). 26701308

[b19] Yan L. . High-Efficiency Genome Editing in Arabidopsis Using YAO Promoter-Driven CRISPR/Cas9 System. Molecular Plant 8, 1820–1823, doi: 10.1016/j.molp.2015.10.004 (2015). 26524930

[b20] Schwartz C. M., Hussain M. S., Blenner M. & Wheeldon I. Synthetic RNA Polymerase III Promoters Facilitate High-Efficiency CRISPR-Cas9-Mediated Genome Editing in Yarrowia lipolytica. ACS synthetic biology, doi: 10.1021/acssynbio.5b00162 (2016). 26714206

[b21] Carrington B., Varshney G. K., Burgess S. M. & Sood R. CRISPR-STAT: an easy and reliable PCR-based method to evaluate target-specific sgRNA activity. Nucleic acids research 43, e157, doi: 10.1093/nar/gkv802 (2015). 26253739 PMC4678847

[b22] Renaud J.-B. . Improved Genome Editing Efficiency and Flexibility Using Modified Oligonucleotides with TALEN and CRISPR-Cas9 Nucleases. Cell Reports 14, 2263–2272, doi: 10.1016/j.celrep.2016.02.018 (2016). 26923600

[b23] Fujii H. . Efficient Multiple Genome Modifications Induced by the crRNAs, tracrRNA and Cas9 Protein Complex in Zebrafish. PloS one 10, e0128319, doi: 10.1371/journal.pone.0128319 (2015). 26010089 PMC4444095

[b24] Sung Y. H. . Highly efficient gene knockout in mice and zebrafish with RNA-guided endonucleases. Genome research 24, 125–131, doi: 10.1101/gr.163394.113 (2014). 24253447 PMC3875853

[b25] Fenghua Z. . A comparison of the knockout efficiencies of two codon-optimized Cas9 coding sequences in zebrafish embryos. Yi chuan = Hereditas/Zhongguo yi chuan xue hui bian ji 38, 144–154, doi: 10.16288/j.yczz.15-452 (2016). 26907778

[b26] Jao L., Wente S. R. & Chen W. Efficient multiplex biallelic zebrafish genome editing using a CRISPR nuclease system. Proceedings of the National Academy of Sciences of the United States of America 110, 13904 (2013). 23918387 10.1073/pnas.1308335110 PMC3752207

[b27] Hwang W. Y. . Heritable and precise zebrafish genome editing using a CRISPR-Cas system. PloS one 8, e68708, doi: 10.1371/journal.pone.0068708 (2013). 23874735 PMC3706373

[b28] Dong Z., Dong X., Jia W., Cao S. & Zhao Q. Improving the efficiency for generation of genome-edited zebrafish by labeling primordial germ cells. The international journal of biochemistry & cell biology 55, 329–334, doi: 10.1016/j.biocel.2014.08.020 (2014). 25194339

[b29] Sakamoto A. . Effect of modification of ovary preservation solution by adding glucose on the maturation and development of pig oocytes after prolonged storage. The Journal of reproduction and development 52, 669–674 (2006). 16873990 10.1262/jrd.17112

[b30] Suttirojpattana T. . The effect of temperature during liquid storage of *in vitro*-matured bovine oocytes on subsequent embryo development. Theriogenology 85, 509–518.e501, doi: 10.1016/j.theriogenology.2015.09.033 (2016). 26483307

[b31] Lee S. H. & Park C. K. Effect of magnetized extender on sperm membrane integrity and development of oocytes *in vitro* fertilized with liquid storage boar semen. Animal reproduction science 154, 86–94, doi: 10.1016/j.anireprosci.2014.12.015 (2015). 25592860

[b32] Cobo A., Garrido N., Pellicer A. & Remohi J. Six years’ experience in ovum donation using vitrified oocytes: report of cumulative outcomes, impact of storage time, and development of a predictive model for oocyte survival rate. Fertility and sterility 104, 1426–1434.e1421–1428, doi: 10.1016/j.fertnstert.2015.08.020 (2015). 26353081

[b33] De Munck N., Belva F., Van de Velde H., Verheyen G. & Stoop D. Closed oocyte vitrification and storage in an oocyte donation programme: obstetric and neonatal outcome. Human reproduction (Oxford, England), doi: 10.1093/humrep/dew029 (2016). 26936884

[b34] Urquiza M. F. . Successful live birth from oocytes after more than 14 years of cryopreservation. Journal of assisted reproduction and genetics 31, 1553–1555, doi: 10.1007/s10815-014-0318-9 (2014). 25205204 PMC4389934

[b35] Yasui G. S. . Improvement of gamete quality and its short-term storage: an approach for biotechnology in laboratory fish. Animal : an international journal of animal bioscience 9, 464–470, doi: 10.1017/s1751731114002511 (2015). 25391393

[b36] Sanches E. A. . Storage of Steindachneridion parahybae oocytes at different temperatures. Animal reproduction science 151, 262–268, doi: 10.1016/j.anireprosci.2014.09.022 (2014). 25458322

[b37] Goetz F. W. & Coffman M. A. Storage of unfertilized eggs of rainbow trout (Oncorhynchus mykiss) in artificial media. Aquaculture 184, 267–276 (2000).

[b38] Ubilla A., Valdebenito I., Arias M. E. & Risopatron J. Viability and DNA fragmentation of rainbow trout embryos (Oncorhynchus mykiss) obtained from eggs stored at 4 degrees C. Theriogenology 85, 1499–1506, doi: 10.1016/j.theriogenology.2016.01.012 (2016). 26893166

[b39] Seki S. . Development of a reliable *in vitro* maturation system for zebrafish oocytes. Reproduction 135, 285–292, doi: 10.1530/REP-07-0416 (2008). 18299421

[b40] Nair S., Lindeman R. E. & Pelegri F. *In vitro* oocyte culture-based manipulation of zebrafish maternal genes. Developmental dynamics : an official publication of the American Association of Anatomists 242, 44–52, doi: 10.1002/dvdy.23894 (2013). 23074011 PMC3857710

[b41] Kimmel C. B., Ballard W. W., Kimmel S. R., Ullmann B. & Schilling T. F. Stages of embryonic development of the zebrafish. Developmental dynamics: an official publication of the American Association of Anatomists 203, 253–310, doi: 10.1002/aja.1002030302 (1995). 8589427

[b42] Westerfield W. M. The zebrafish book: a guide for the laboratory use of zebrafish (Danio rerio). (University of Oregon press, 2000).

[b43] Wang J. W. . CRISPR/Cas9 nuclease cleavage combined with Gibson assembly for seamless cloning. BioTechniques 58, 161–170, doi: 10.2144/000114261 (2015). 25861928

[b44] Borges A. . CTAB methods for DNA extraction of sweetpotato for microsatellite analysis. Scientia Agricola 66, 529–534 (2009).

[b45] Starkings S. Quantitative Data Analysis with IBM SPSS 17, 18 & 19: A Guide for Social Scientists by Alan Bryman and Duncan Cramer. International Statistical Review 80, 334–335 (2012).

[b46] Chen F. . High-frequency genome editing using ssDNA oligonucleotides with zinc-finger nucleases. Nature methods 8, 753–755, doi: 10.1038/nmeth.1653 (2011). 21765410 PMC3617923

[b47] Kimura Y., Hisano Y., Kawahara A. & Higashijima S. Efficient generation of knock-in transgenic zebrafish carrying reporter/driver genes by CRISPR/Cas9-mediated genome engineering. Scientific reports 4, 6545, doi: 10.1038/srep06545 (2014). 25293390 PMC4189020

[b48] Nakajima K. & Yaoita Y. Highly efficient gene knockout by injection of TALEN mRNAs into oocytes and host transfer in Xenopus laevis. Biology open 4, 180–185, doi: 10.1242/bio.201410009 (2015). 25596277 PMC4365486

[b49] Hwang W. Y. . Efficient genome editing in zebrafish using a CRISPR-Cas system. Nature biotechnology 31, 227–229, doi: 10.1038/nbt.2501 (2013). PMC3686313 23360964

[b50] Gagnon J. A. . Efficient mutagenesis by Cas9 protein-mediated oligonucleotide insertion and large-scale assessment of single-guide RNAs. PloS one 9, e98186, doi: 10.1371/journal.pone.0098186 (2014). 24873830 PMC4038517

