# Supplementary material for: A novel technique based on in vitro oocyte injection to improve CRISPR/Cas9 gene editing in zebrafish
Source: Sci Rep. 2016 Sep 29;6:34555. doi: 10.1038/srep34555 (PMC5041118; doi:10.1038/srep34555)
Supplement: Supplementary Information [file srep34555-s1.pdf]

Supporting information

A novel technique based on *in vitro* oocyte injection to improve CRISPR/Cas9 gene editing in zebrafish

Shao-Lin Xie[1, 2], Wan-Ping Bian[2], Chao Wang[1, 2], Muhammad Junaid[2, 3], Ji-Xing Zou[1*], De-Sheng Pei[2*]

[1]College of Marine Sciences, South China Agricultural University, Guangzhou, 510642, China

[2]Chongqing Institute of Green and Intelligent Technology, Chinese Academy of Sciences, Chongqing, 401122, China

[3]University of Chinese Academy of Sciences, Beijing 100049, China

[*]Corresponding authors.

E-mails:zoujixing@163.com(J.X.Z), deshengpei@gmail.com (D.S.P) and peids@cigit.ac.cn (D.S.P)

Table S1 Toxicity of Cas9 capped RNAs and sgRNAs in oocytes*

	Total amount			Fertilization			Hatching		
Trials No.	1	2	3	1	2	3	1	2	3
Control	104	112	105	83	87	81	79	85	75
Phenol red	111	124	116	68	74	60	63	69	55
Cas9/sgRNA	118	135	120	72	71	58	58	55	44

*Control represented oocytes stored *in vitro* for 30 min without injection. Phenol red or Cas9/sgRNA indicated that oocytes were stored *in vitro* for 30 min and then injected with phenol red or Cas9 capped RNAs and sgRNAs, respectively.

Table S2 The efficiency of *mloxP* knocked into *mc4r* in zebrafish*

	Amount of detection	Amount of knock-in
Oocytes storage injection	25	13
	30	15
	30	14
Normal injection	25	7
	30	8
	30	7

*The experiment was repeated three times. The larvae carrying *mloxP* were detected by PCR at 72 hpf with *mc4r T7E* forward primer matching *mc4r* sequence and the *mloxP* reverse primer matching the sequence of *mloxP* donor sequence.

Table S3 The amount of mutations in P_0 generation using two different methods*

Number of detection	The amount of mutation									
	mc4r		***mpv17***		***mrap2b***		***mc3r***		***mstna***	
	**nP_0**	**sP_0**	**nP_0**	**sP_0**	**nP_0**	**sP_0**	**nP_0**	**sP_0**	**nP_0**	**sP_0**
30	25	28	5	26	14	28	10	27	7	27
30	26	28	7	27	12	29	11	28	11	27
30	25	29	5	27	11	27	9	26	11	28

*For each gene, the genomic DNAs were extracted from zebrafish tail at random and measured by T7E1 assay. The results of T7E1 assay were confirmed by sequencing analysis. nP_0 and sP_0 represented the generations from normal injected zygotes and injected oocytes after storage, respectively.

Table S4 The efficiencies of germline transmission of *mc4r* and *mpv17**

No.	Gene		Number of detection	Number of mutations
1	*mc4r*	nF1	20	14
		sF1	28	27
	mpv17	nF1	17	6
		sF1	20	18
2	*mc4r*	nF1	24	16
		sF1	26	26
	mpv17	nF1	21	7
		sF1	25	23
3	*mc4r*	nF1	22	16
		sF1	27	25
	mpv17	nF1	19	7
		sF1	20	18

*nF1 and sF1 represented the embryos from normal injection P_0 and oocytes storage injection P_0, respectively.

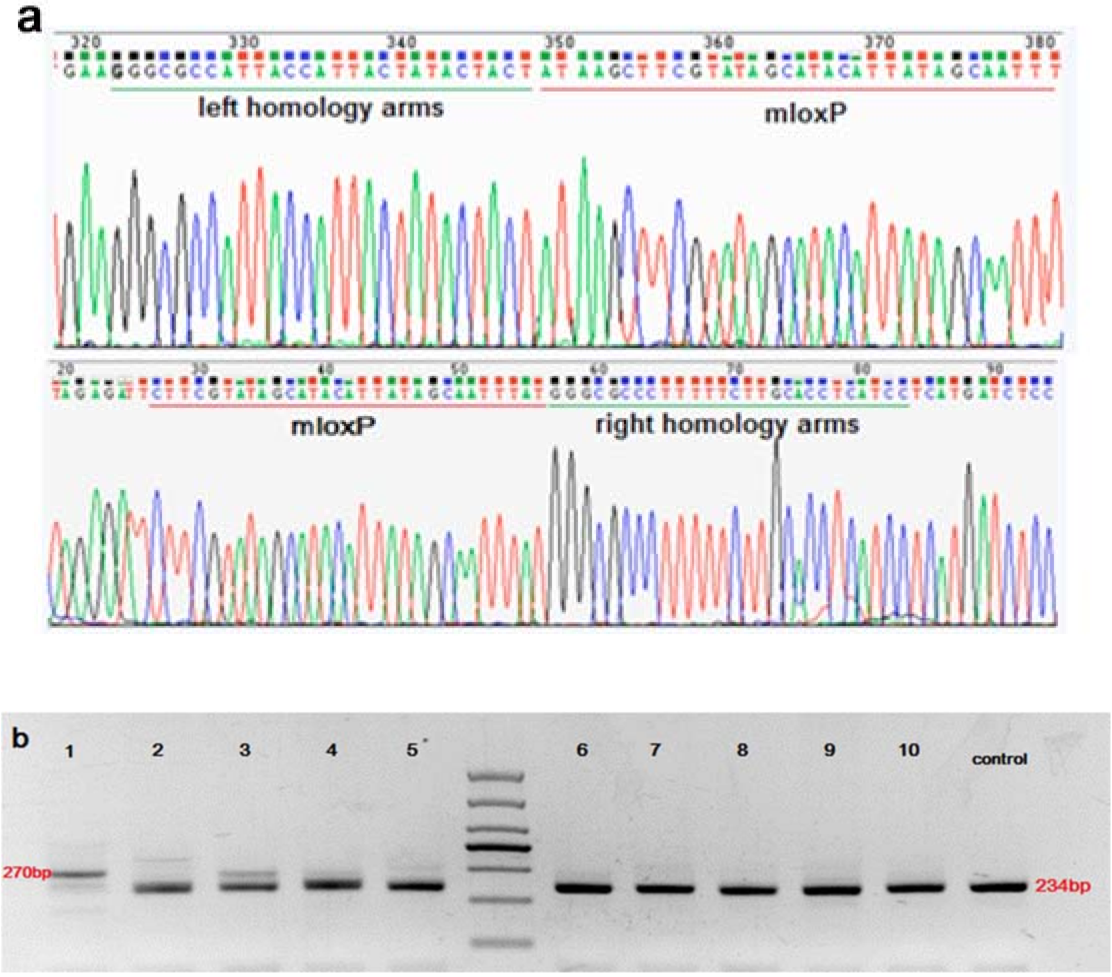

Figure S1. Identification of knocking *mloxP* gene into the *mc4r* locus in zebrafish using the novel technique based on *in vitro* oocyte injection. (a) Sequencing result showed the correct insertion of *mloxP* in the *mc4r* locus. PCR fragments were amplified using primer pairs *mc4r*-T7E-F/*mloxp*-R (upper panel) and *mloxp*-F/*mc4r*-T7E-R (lower panel) from 5 tested embryos for each groups and cloned into the pMD-19T vector for sequencing (4-6 clones/embryo). (b) PCR products from the genomic locus flanking the target site using primer pairs (*mloxP*- KI-JC-F/*mloxP*- KI-JC-R). The WT amplicons were 234 bp but *mloxP* knock-in amplicons were 270 bp. 1 -5: oocyte storage eggs; 6 -10: normal injection eggs.

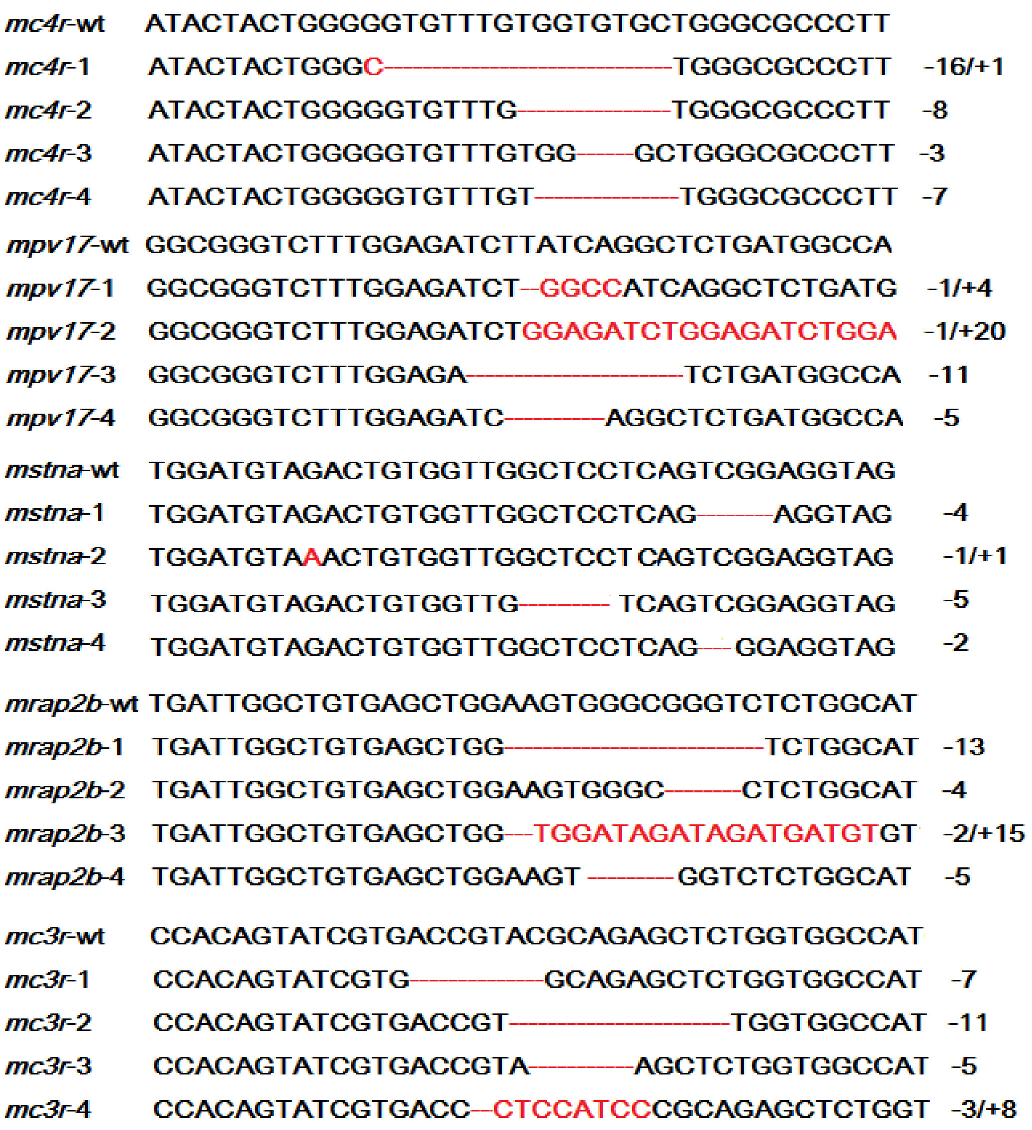

Figure S2. Genomic DNA sequencing of mutations induced by Cas9 cleavage at the targeted *mc4r*, *mpv17*, *mstna*, *mc3r* and *mrap2b* genes. The positive fragments confirmed by T7E1 assay were inserted into the pMD-19T vector and randomly sequenced. The deleted (-) and inserted (+) nucleotides were shown compared to the wild-type.

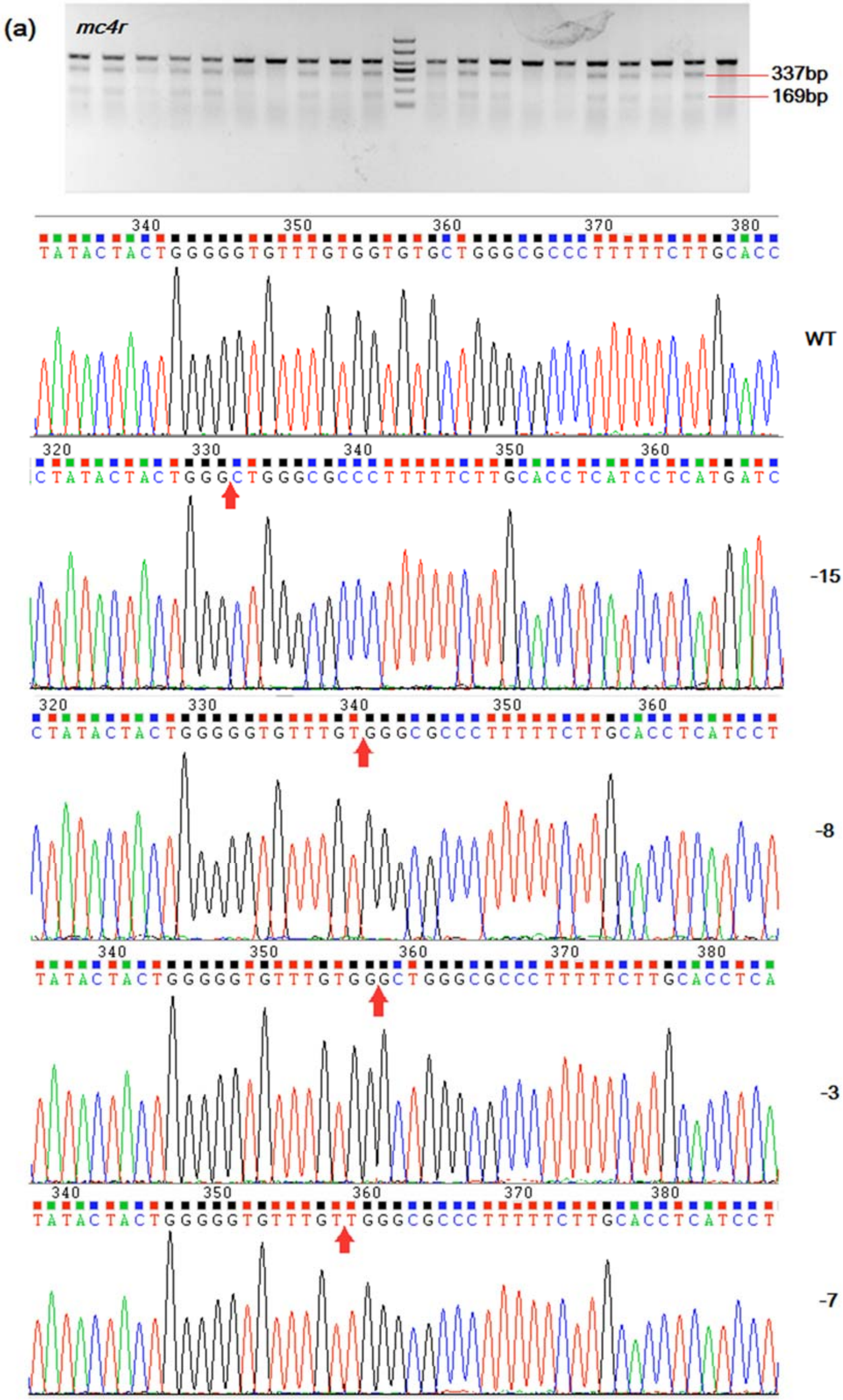
(a)
mc4r
337bp
169bp
WT
-15
-8
-3
-7

(b)

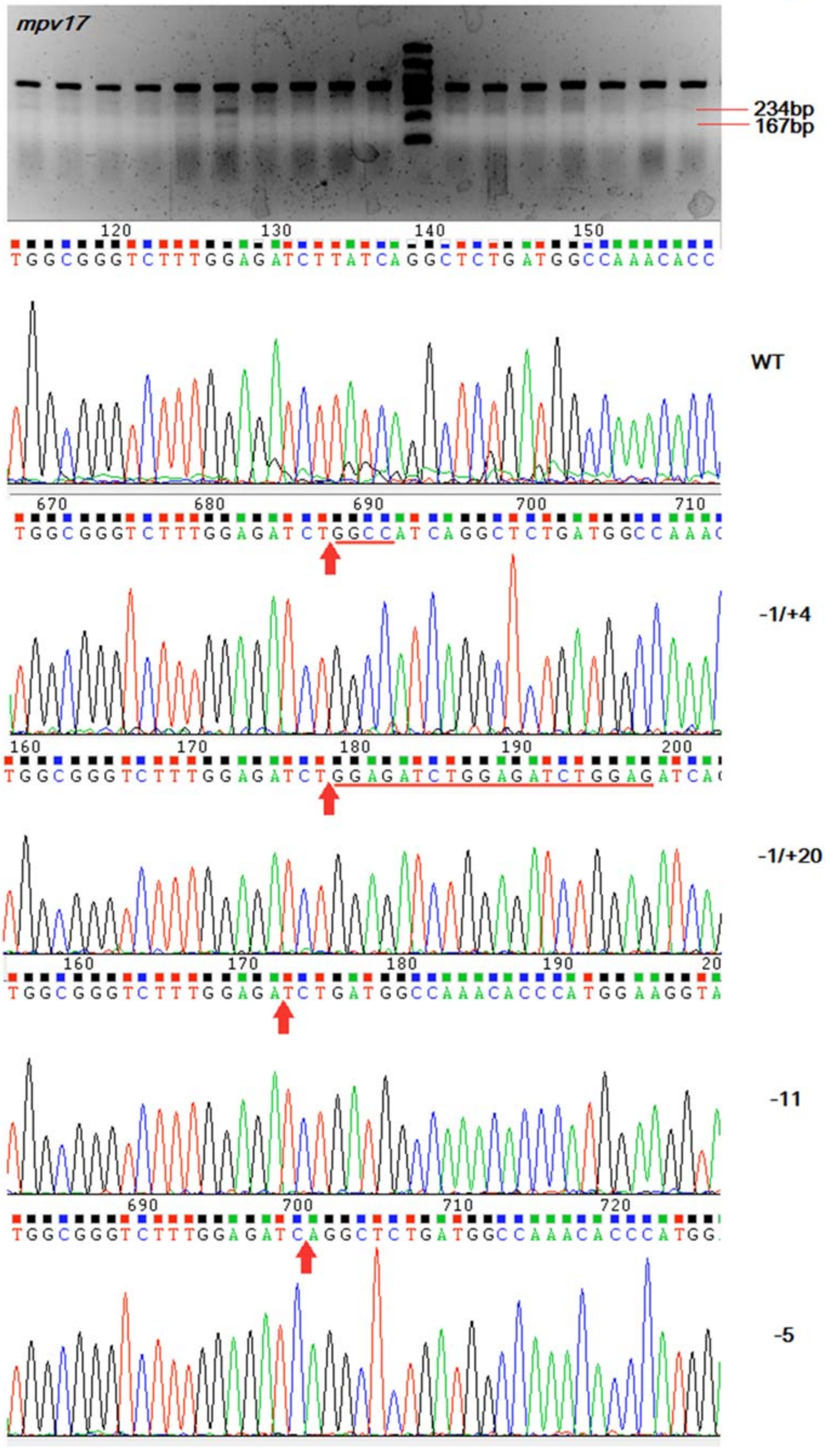
mpv17
234bp
167bp
TGGCGGGTCTTTGGAGATCTTATCAGGCTCTGATGGCCAAACACC
WT
TGGCGGGTCTTTGGAGATCTGGCCATCAGGCTCTGATGGCCAAAC
-1/+4
TGGCGGGTCTTTGGAGATCTGGAGATCTGGAGATCTGGAGATCA
-1/+20
TGGCGGGTCTTTGGAGATCTGATGGCCAAACACCCATGGAAGGTA
-11
TGGCGGGTCTTTGGAGATCAGGCTCTGATGGCCAAACACCCATGG
-5

(c)

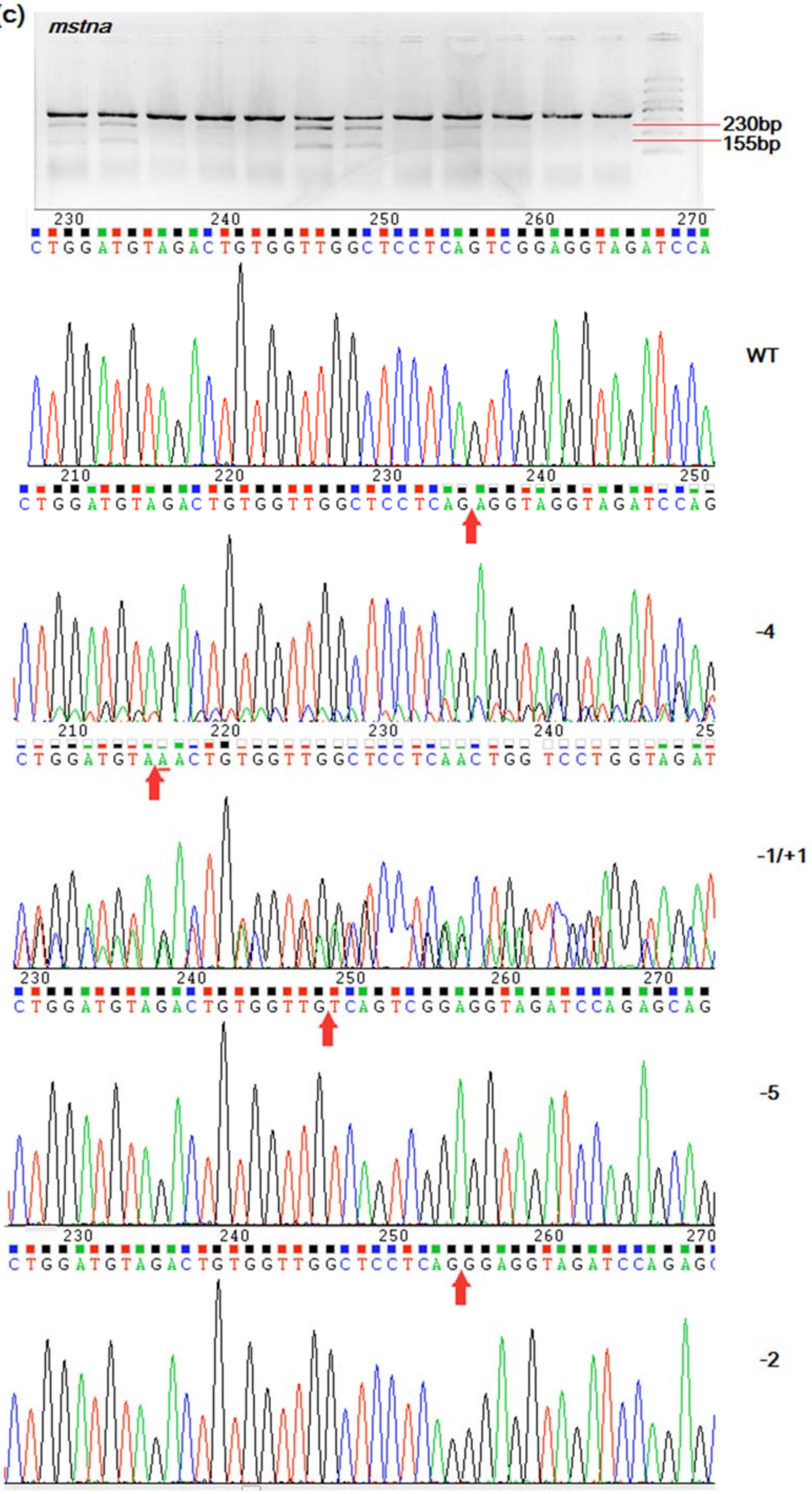

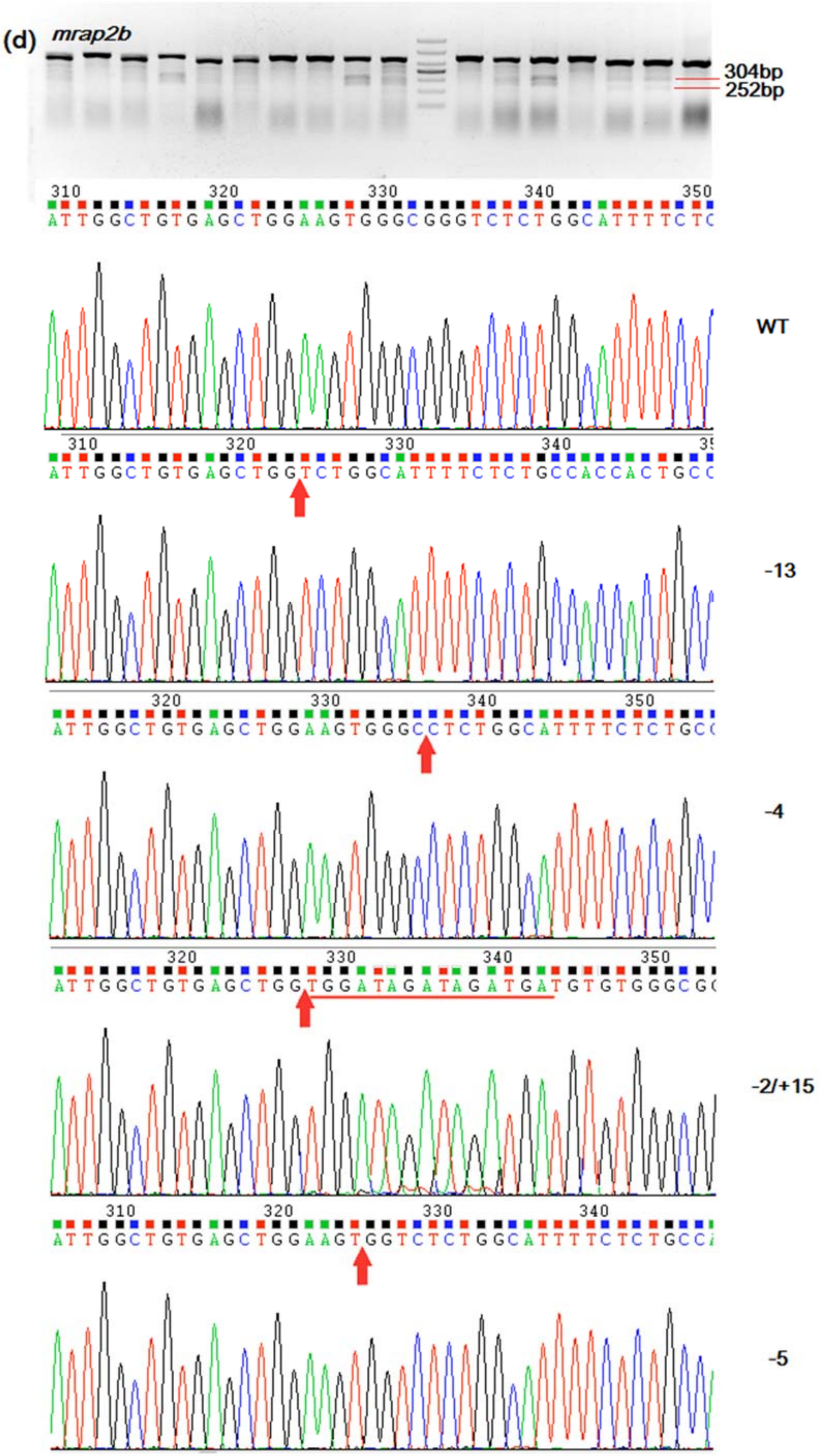
(d)
mrap2b
304bp
252bp
WT
-13
-4
-2/+15
-5

(e)

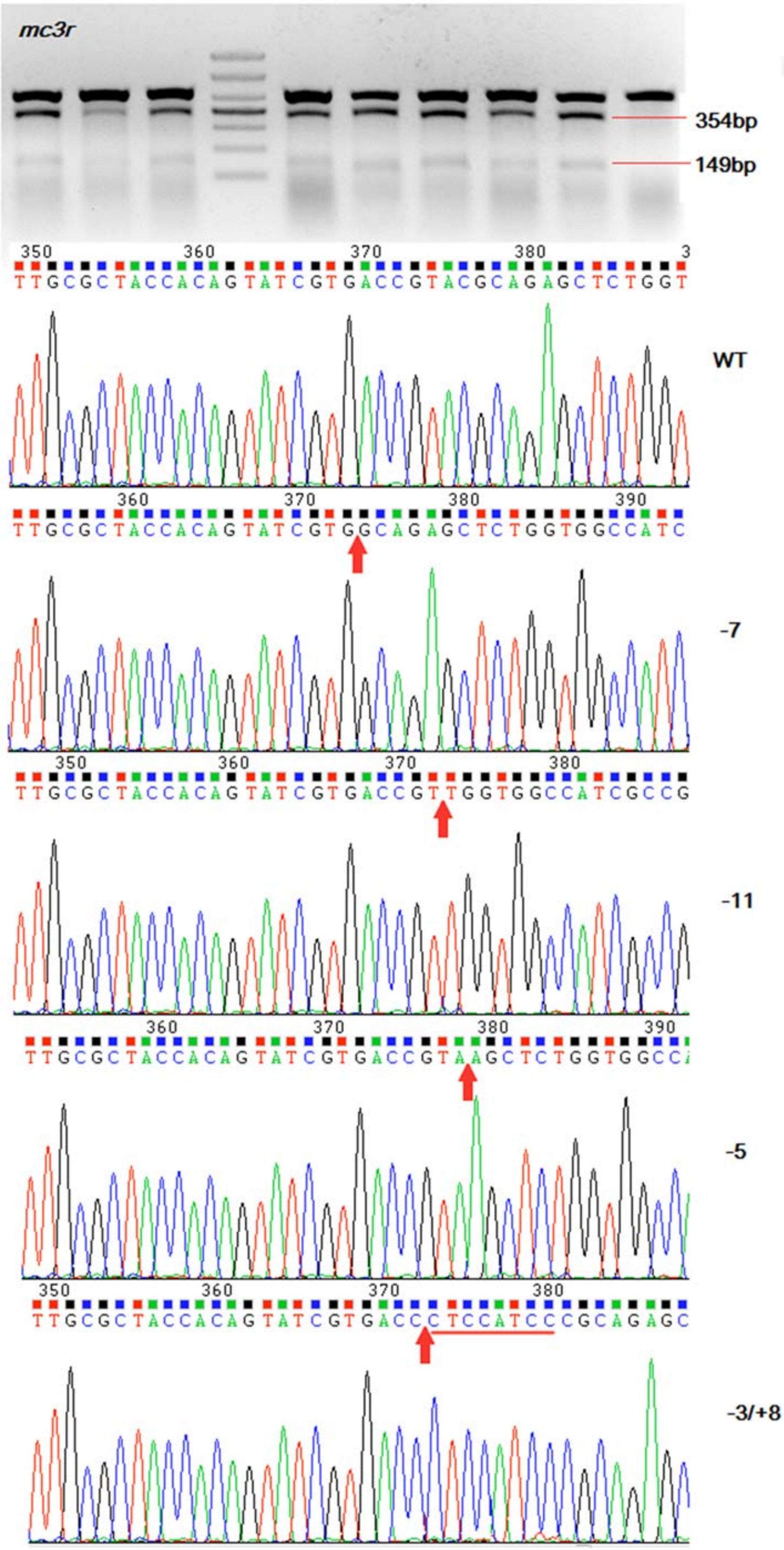

Figure S3. T7E1 assay and DNA sequencing of five genes disrupted by the novel CRISPR-Cas system based on *in vitro* oocyte injection and storage. (a), (b), (c), (d) and (e) were *mc4r*, *mpv17*, *mstna*, *mrap2b* and *mc3r*, respectively.
